# Supplementary material for: Clinical and Analytical Performance of ELISA Salivary Serologic Assay to Detect SARS-CoV-2 IgG in Children and Adults
Source: Antibodies (Basel). 2024 Jan 5;13(1):6. doi: 10.3390/antib13010006 (PMC10801479; doi:10.3390/antib13010006)
Supplement: Supplementary file 1 [file antibodies-13-00006-s001.zip › antibodies-2747558-supplementary.pdf]

Supplementary Table S1: Repeatability performances, estimated using 6 samples

Sample number	Experimental set-up	Mean value (kAU/L)	Repeatability precision (%)
1	Five repetition, randomly pipetted in one plate	1.61	5.5
2	Five repetition, randomly pipetted in one plate	3.77	3.9
3	Five repetition, randomly pipetted in one plate	5.96	5.99
4	Five repetition, randomly pipetted in one plate	7.42	23.6
5	Five repetition, randomly pipetted in one plate	10.1	15.90
6	Twenty-five repetitions in different plates (5 each plate)	2.01	20.9
